# Eight previously unidentified mutations found in the *OA1 *ocular albinism gene

**DOI:** 10.1186/1471-2350-7-41

**Published:** 2006-04-28

**Authors:** Hélène Mayeur, Olivier Roche, Christelle Vêtu, Carolina Jaliffa, Dominique Marchant, Hélène Dollfus, Dominique Bonneau, Francis L Munier, Daniel F Schorderet, Alex V Levin, Elise Héon, Joanne Sutherland, Didier Lacombe, Edith Said, Eedy Mezer, Josseline Kaplan, Jean-Louis Dufier, Cécile Marsac, Maurice Menasche, Marc Abitbol

**Affiliations:** 1 EA no 2502 du ministère de la Recherche, de l'Enseignement Supérieur et la Technologie, CEntre de Recherches Thérapeutiques en Ophtalmologie, (CERTO), Université René Descartes-Paris V, Faculté de Médecine René Descartes-Site Necker, 156 rue de Vaugirard, 75015 Paris cedex, France; 2 Service d'ophtalmologie, Hôpital Necker-Enfants Malades, 149 rue de Sèvres, 75015 Paris, France; 3 Laboratoire de diagnostic génétique, Hôpitaux Universitaires de Strasbourg, Strasbourg, France; 4 Service de génétique Médicale, CHU d'Angers, France; 5 Jules Gonin Eye Hospital, Lausanne, Switzerland; 6 IRO-Institut de Recherche en Ophtalmologie, Sion, Switzerland; 7 Department of Ophthalmology and Vision Sciences, The Hospital for Sick Children, 555 University Avenue, Toronto, Ontario, M5G 1X8, Canada; 8 Service de Génétique Médicale, Hôpital Pellegrin-Enfants, Bordeaux, France; 9 Department of Pediatrics and Medical Genetics, St. Luke's Hospital, Gwardamangia, Malta; 10 Alberto Moscona Department of Ophthalmology, Rambam Health Care Campus, Haifa, Israel; 11 Service de Génétique Médicale du CHU Necker-Enfants Malades, Unité INSERM 393, 149 rue de Sèvres, 75015, Paris, France

## Abstract

**Background:**

Ocular albinism type 1 (OA1) is an X-linked ocular disorder characterized by a severe reduction in visual acuity, nystagmus, hypopigmentation of the retinal pigmented epithelium, foveal hypoplasia, macromelanosomes in pigmented skin and eye cells, and misrouting of the optical tracts. This disease is primarily caused by mutations in the *OA1 *gene.

**Methods:**

The ophthalmologic phenotype of the patients and their family members was characterized. We screened for mutations in the *OA1 *gene by direct sequencing of the nine PCR-amplified exons, and for genomic deletions by PCR-amplification of large DNA fragments.

**Results:**

We sequenced the nine exons of the *OA1 *gene in 72 individuals and found ten different mutations in seven unrelated families and three sporadic cases. The ten mutations include an amino acid substitution and a premature stop codon previously reported by our team, and eight previously unidentified mutations: three amino acid substitutions, a duplication, a deletion, an insertion and two splice-site mutations. The use of a novel Taq polymerase enabled us to amplify large genomic fragments covering the *OA1 *gene. and to detect very likely six distinct large deletions. Furthermore, we were able to confirm that there was no deletion in twenty one patients where no mutation had been found.

**Conclusion:**

The identified mutations affect highly conserved amino acids, cause frameshifts or alternative splicing, thus affecting folding of the OA1 G protein coupled receptor, interactions of OA1 with its G protein and/or binding with its ligand.

## Background

Ocular albinism type 1 (Nettleship-Falls type, OMIM +300500) is the most common form of ocular albinism. The disease is transmitted as an X-linked disorder, in which affected males show all the ocular signs of albinism: severely impaired visual activity, nystagmus, photophobia, iris transillumination, hypopigmentation of retinal pigmented epithelium, foveal hypoplasia and misrouting of optic tracts. Most female carriers (90%) show minor fundal signs, suggesting a potential carrier status. Most of the affected patient'mothers present a typical mosaic pattern of depigmentation assumed to represent the effect of random X-inactivation (Lyonization) of the *OA1 *gene. However, in some mothers the depigmentation pattern looks very subtle and may correspond to normal fundoscopic aspects.

The *OA1 *gene (*GPR143*, [GenBank NM_000273]) is expressed in skin and retinal pigmented epithelial cells. In these cells, the melanosomes are larger than normal melanosomes [1,2]. The OA1 protein is a G protein-coupled receptor (GPCR) that is embedded in the melanosome membrane [3], with the N-terminus of the protein in the melanosome lumen and the C-terminus in the cytosol. OA1 is the only known intracellular GPCR and its signaling pathway is involved in regulating melanosome biogenesis and growth [4,5]. The OA1 ligand, which is presumably present inside the melanosome, remains unknown [6].

Ocular albinism is primarily caused by mutations in the *OA1 *gene [7]. The *OA1 *gene spans about 40 kb of genomic DNA in chromosome Xp22.3 and contains nine exons [8]. Many different mutations have been reported, some of which cause abnormal folding of the protein. Other mutations affect the receptor interaction with its G protein, thus altering the signaling pathways, or with its ligand [9]. So far, 44 mutations and 18 deletions have been discovered [10,11]. However, no mutations have been reported in the C-terminal end of the protein.

We screened 72 individuals, representing 37 probands, and identified mutations in seven unrelated families and three patients with sporadic disease. We found eight previously unidentified mutations: three missense mutations, a duplication, a deletion, an insertion and two splice-site mutations. We also found two mutations previously reported by our team: one missense and one nonsense mutation [12]. These ten mutations were named using the international nomenclature [13]. Nucleotide numbering used the A of the ATG start codon as nucleotide +1. Moreover, we detected six probable deletions which remain to be characterized.

## Methods

### Patients

The study population consisted of 72 individuals, representing 37 probands. 39 of these individuals were affected by ocular albinism, 27 were carriers and 6 were unaffected individuals. We included in this study 100 ethnically matched control individuals not affected by any form of albinism. We analyzed the exon sequences and the intron/exon boundaries of the *OA1 *gene in all 72 subjects. Whenever possible, affected males and their families underwent clinical ophthalmologic examination, including visual acuity testing, fundus examination and multichannel visual evoked potential analysis to confirm X-linked ocular albinism. Blood samples were collected from patients after signed informed consent by the adults or by both parents for each child involved in the study. We strictly obeyed the rules of bioethical laws of the European Union and France, as well as the guidelines of the Helsinki Declaration.

### DNA amplification and sequencing

We extracted genomic DNA from the lymphocytes of all subjects, and amplified all nine exons from all the samples by PCR using intronic oligonucleotide primers. We generated two overlapping PCR fragments (A for upper fragment and B for lower fragment) for exons 1, 8 and 9, because of their length. The primer sequences were:

1AF-GAGCCTGGCTCTACTGCAGGCGCTG, 1AR-CCTTCCACGCGCTCTGGCT, 1BF-AGCCACGCAGCTCGTGCTGAGCTTCCAGCC, 1BR-CCGCGGGTTGGAATCTGATCAGCGCCTGGG, 2F-TGATTAGGATCAGATACAAAG, 2R-CCTACTTATGCTCCTCAAAG, 3F-TTCTTGTACCTGTTTCCAGAC, 3R-CAAGATAAGAGATGGCACTGA, 4F-AGTTCCAGGCAGGCCTCTGT, 4R-TCTTGCAGGGAATACATGAGC, 5F-CATCCTCTTATCTTGACTTCC, 5R-ACTAGGGAAGCTCTCTGGG, 6F-CTGTCACTCCGTAAACATGAA, 6R-CCAGTGGGAAGGGCTATGGAA, 7F-CATGTTCTCTTTACCTGCTGC, 7R-CTTCAGAGACAAGGTCTCACT, 8AF-CTGCACTGACTGCCATGTGTC, 8AR-GAATCACTGACCACCTCGGCT, 8BF-CAGTCTCCCAGGAAGGAGATC, 8BR-GAAGTCTACCTGCTGTGGCAG, 9AF-AGCTGATGACAAACCTGCTAG, 9AR-CTTTAGGATAGGAGAAAGGG, 9BF-CCCATATTCCTCAGACTCAAC, 9BR-TGGATTCTGTCTTAACACTTC.

The conditions of amplification of each fragment are available upon request. The DNA fragments were then purified on NucleoFast plates (Macherey-Nagel) and the sense and antisense strands were sequenced on an ABI 777 Machine (Applied Biosystems) by the INSERM core automated sequencing facility of Hôpital Cochin, Paris.

### Splice site mutations

We analyzed sequences that could contain nucleotide changes that could affect the splicing of the *OA1 *gene using NNSPLICE 0.9 software [14] to search for potential abnormal new splice sites generated by mutations.

### Sequence aligments

The OA1 protein was then aligned with its orthologs and other GPCRs using BioEdit software [15] and other online software, such as Pfam [16], to determine the conservation of modified amino acids between species and among GPCRs.

### Deletions

The *OA1 *gene was screened for deletions by PCR on genomic DNA, using TaKaRa LA Taq enzyme (Cambrex). We divided *OA1 *in three fragments, 15 kb each. The primer sequences used for the long-rang amplifications were:

OA1-1-F: CCCAAATAGGAAAGATCTAGTCCACGAGGC,

OA1-1-R: ACTTACTCCTCTTACAGCCTCATCCCAGGA,

OA1-2-F: GCAGGCTTGAGCTCAGGAGTTTGAAGTTAC,

OA1-2-R: GCCTGGTAGAGTTTGGGGCAGTCATAAGTA,

OA1-3-F: CCACCAGCTGGAACTGCTGTTAGTCTGTAG,

OA1-3-R: CTGTCTTCTCTGCCCCCTAGTGTGTGTTAG.

The conditions of amplification of each fragment are available upon request.

We also looked carefully at the PCR electrophoresis gels as well as the chromatograms, in order to detect which exons could be deleted.

## Results

After ophthalmological examination, we found that the visual acuity of all affected individuals was severely impaired compared to age-, sex- and refractive error-matched normal patients. Female carriers had a mosaic depigmentation pattern of their fundus which could be related to X chromosome inactivation (Lyonization). Control patients had no abnormality of their skin and eye pigmentation.

**Figure 1:**
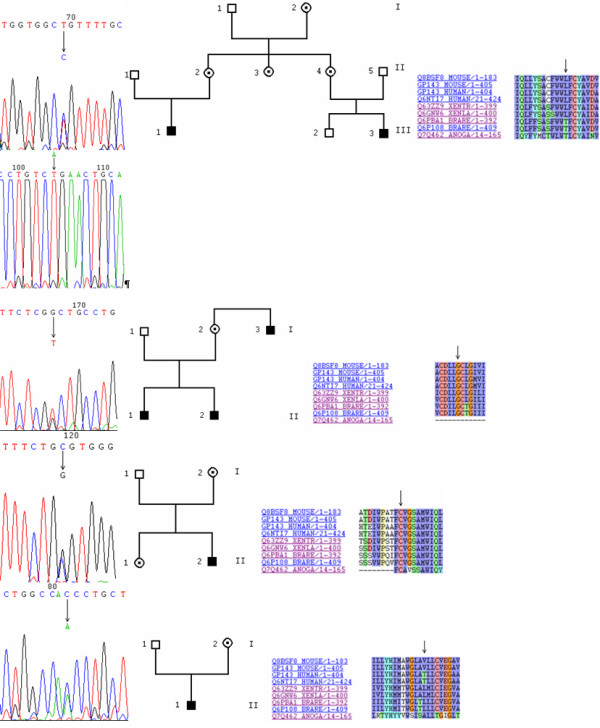
point mutations. 1A: chromatogram of the 401T>C mutation (L134P), from a carrier, obtained with Chromas 2.3. The nucleotide change resulting from the mutation is shown on the chromatogram. 1B: pedigree of the whole family with its three branches: solid squares are affected people, dotted circles are molecularly proven carriers and hollow squares and circles are unaffected people. Here, I2 and II 1, 2 and 3 are carriers, III1 and 3 are affected. The mutation was previously described in II3 and III3 [12]. 1C: sequence alignment of OA1 between different species: MOUSE: Mus musculus, HUMAN: Homo sapiens, XENTR: Xenopus tropicalis, XENLA: Xenopus laevis, BRARE: Danio rerio, ANOGA: Anopheles gambiae. The arrows in the alignment show the amino acid affected by the mutation. 1D: chromatogram of the 853A->T mutation (R285X), from an affected individual. 1E: chromatogram of the 241G>T mutation (G81V), from an affected individual. 1F: pedigree of the family affected by G81V. 1G: sequence alignment around G81. 1H: chromatogram of the 348C>G mutation (C116W), from a carrier. 1I: pedigree of the family affected by C116W. 1J: sequence alignment around C116. 1K: chromatogram of the 497C>A mutation (T166N), from an affected individual. 1L: pedigree of the family affected by T166N. 1M: sequence alignment around T166.

**Figure 2:**
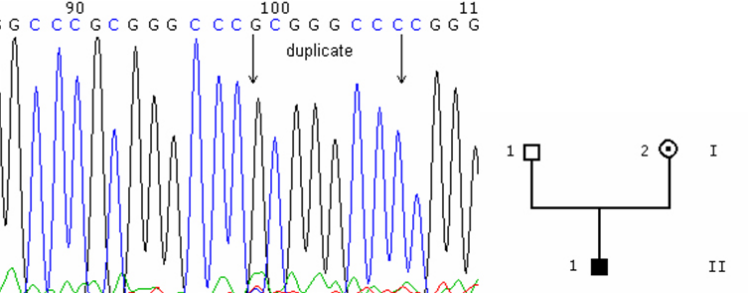
duplication. 2A: chromatogram of the 163_170dup(GCGGGCCC) mutation, from an affected individual. 2B: pedigree.

**Figure 3:**
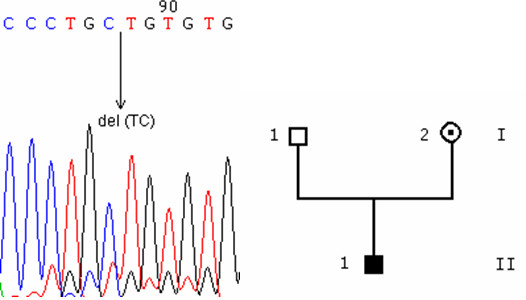
deletion. 3A: chromatogram of the 504_505del(CT)mutation, from an affected individual. 3B: pedigree.

**Figure 4:**
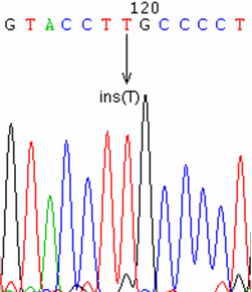
insertion. 4A: chromatogram of the 601_602ins(T) mutation, from an affected individual.

**Figure 5:**
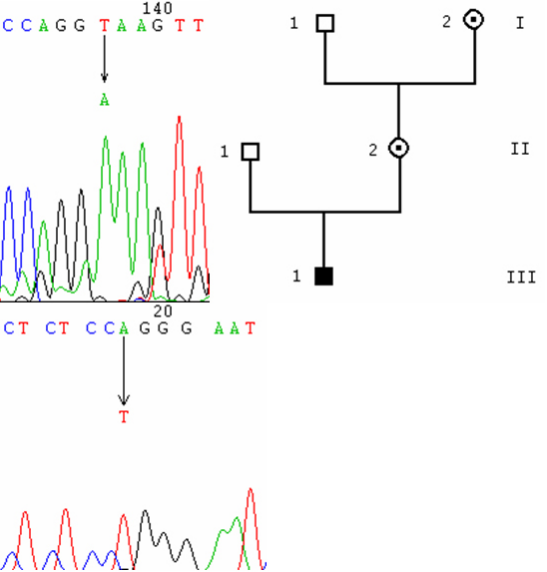
splice site mutations. 5A: chromatogram of the 547+2T->A mutation, from an affected individual. 5B: pedigree of the family affected by the 547+2T->A mutation. 5C: chromatogram of the 886-2A->T mutation, from an affected individual.

The molecular genetics results are summarized in Table 1 and shown in Figures 1, 2, 3, 4, 5. We found two previously described mutations [12]. The first nucleotidic change, c.401T>C (p.L134P amino acid change, Figure 1A, 1B, 1C), located in exon 3, was found in two related families. In one family, I2 and her daughter II3 carry the mutation in the heterozygous state, whereas in the other family, II2, who turned out to be the sister of II3, is heterozygous whereas her son, III1, is hemizygous. In a third branch previously reported by our team [12], II4, found to be the other sister of II3, is heterozygous whereas her son, III3, is hemizygous. The second already found nucleotidic mutation, the R285X change (c.853A>T, Figure 1D), located in exon 7, was found in another patient which was apparently a sporadic case.

**Table 1:** Mutations of *OA1*. The amino acid changes were deduced from the genomic sequence.

Patient	Nucleotide change	Amino acid change	Exon	Protein domain	Reference
A (familial case)	401T->C	L134P	3	TMIII	[12]
B (Canadian sporadic case)	853A->T	R285X	7	l3	[12]
C (French familial case)	241G->T	G81V	1	TMII	Previously unidentified mutation
D (Canadian sporadic case)	348C->G	C116W	2	l1	Previously unidentified mutation
E (French sporadic case)	497C->A	T166N	4	TMIV	Previously unidentified mutation
F (French sporadic case)	163_170dupGCGGGCCC	G58fsX29 (Frameshift)	1	i1	Previously unidentified mutation
G (French sporadic case)	504_505delCT	L168fsX58 (Frameshift)	4	TMIV	Previously unidentified mutation
H (Canadian sporadic case)	601_602insT	P201fsX25 (Frameshift)	5	TMV	Previously unidentified mutation
I (Canadian sporadic case)	547+2T->A	Splice site mutation	4	TMIV	Previously unidentified mutation
J (Canadian sporadic case)	886-2A->T	Splice site mutation	8	l3	Previously unidentified mutation

We also found eight previously unidentified mutations in six other unrelated families and two other isolated cases.

We observed a c.241G>T mutation (p.G81V, Figure 1E, 1F, 1G) located in exon 1 in two affected brothers (II1 and II2) as well as in their uncle (I3) in the hemizygous state and in their mother (I2) in the heterozygous state.

We detected a c.348C>G mutation (p.C116W, Figure 1H, 1I, 1J) located in exon 2 in a male patient (II2) in the hemizygous state and in his sister (II1) and mother (I2) in the heterozygous state.

We found a c.497C>A mutation (p.T166N, Figure 1K, 1L, 1M) located in exon 4 in a male patient (II1) in the hemizygous state and in his mother (I2) in the heterozygous state.

We found an 8-base pair duplication (c.163_170dupGCGGGCCC, Figure 2) located in exon 1 in an affected male (II1) in the hemizygous state, and in his mother (I2) in the heterozygous state.

We also found a 2-base pair deletion (c.504_505delCT, p.L168fsX58, Figure 3) located in exon 4 in an affected male (II1) in the hemizygous state, and his mother (I2) in the heterozygous state, and a 1-base pair insertion (c.601_602insT, p.P201fsX25, Figure 4) located in exon 5 in a sporadic case.

We found two splice site mutations. One of these is located just after exon 4 (c.547+2T>A, Figure 5A, 5B), in the 5' consensus donor region for the splicing of intron 4–5. This mutation was found in the hemizygous state in an affected patient (III1), and in the heterozygous state in his mother (II2) and his maternal grandmother (I2).

The other splice site mutation (c.886-2A>T, Figure 5C), located in the 3' consensus acceptor region for the splicing of intron 7–8, was found in an isolated case.

The *OA1 *gene from all the control individuals included in this study was sequenced, and we found none of the mutations that we detected in the *OA1 *gene sequences.

The 27 patients who apparently did not carry any *OA1 *mutation in our initial mutational screening were screened for large genomic deletions in the *OA1 *gene. The use of the TaKaRa Taq enzyme combined with the analysis of the gels and chromatograms led us to conclude that twenty-one patients did not to carry any deletion. We are working on three familial cases and three isolated patients who present abnormal amplification of the gene (data not shown) but their genetic alterations are not characterized yet.

## Discussion

We studied 39 individuals with ocular albinism and members of their families. We found mutations in 12 individuals out of 39 (31%) with a clinical diagnosis of ocular albinism. We found two previously reported mutations and eight novel sequence changes in the *OA1 *gene. Among the previously reported mutations, the L134P change was found in what seemed to be three unrelated French families. These families were initially examined in two different countries, by three different ophthalmologists and three different medical geneticists. Details about the extended family were either overlooked or not known. The same nucleotide mutation (401T>C) was not found in 100 ethnically matched control patients. From a meticulous study by interview of the patients' pedigree, it was found that these families are three branches of the same family (Figure 1B). The unmarried lone daughter (II3) of one family and the mothers of the other two families (II2 and II4) are in fact three sisters who had not readily disclosed their biological familial links. Sequence alignment analysis showed that L134 is conserved among all tetrapod OA1 proteins. This result suggests that this amino acid may play a key role in OA1 protein function. Therefore a mutation of this leucine residue, located in the third transmembrane domain, may result in a non-functional protein and explains the severe visual phenotype observed in the affected patients.

The mutation R285X resulted in the OA1 protein being truncated in its third luminal loop.

The G81V mutation affects a glycine residue conserved among the vertebrate orthologs of OA1. This valine residue, located in the second transmembrane domain, is larger than the glycine residue and may prevent the protein from folding correctly.

The C116W mutation affects a cysteine residue that is highly conserved between all species and among all GPCRs. This cysteine is thought to form a disulfide bond with C184, allowing the correct folding of the receptor. The mutation modifies the protein in the very end of the first luminal loop. Thus, the protein cannot fold properly and is therefore completely non-functional [17]. Two other mutations have been reported to affect this amino acid: C116S and C116R [9,18].

Alignments of all the vertebrate OA1 proteins show that a hydrophobic amino acid is found in most vertebrate OA1 proteins at the position corresponding to the human OA1 amino acid residue 166, which is normally a threonine (figure 1M). Although threonine and asparagine are both polar amino acids, threonine is much less hydrophilic than asparagine which is the mutated amino acid. Moreover, substituting the threonine at codon 166 with an asparagine substantially changes the residue side chain at a critical position of the fourth transmembrane domain, in the OA1 protein. Therefore, this amino acid substitution very likely causes structural and functional alterations to OA1 and is thus very likely to be highly pathogenic.

The c.163_170dupGCGGGCCC duplication is located in the region encoding the first intracellular loop and induces a frameshift and a premature stop codon (p.G58fsX29). This truncates the protein in its second transmembrane domain, completely preventing any function of the OA1 protein.

Both c.504_505delCT and c.601_602insT mutations induce a frameshift and a premature stop codon, truncating the protein in its third intracellular loop.

We used NNSPLICE in order to analyze the mutated mRNAs resulting from the splice site mutations, and we found that they had displaced splice sites, with neighboring cryptic splice sites being used instead of the missing normal splice site. This resulted in short erratic sequences ending with a stop codon after the normal sequence. Mechanistically, this observation is somewhat reminiscent of that previously reported by our lab in a case of pyruvate dehydrogenase deficiency [19]. Thus, if the abnormal mRNA are actually translated and escape degradation, the encoded proteins are truncated and non-functional.

Recent findings have shown that new splice sites can appear within introns, leading to aberrant mRNA [19,20]. These splice sites cannot be found at the genomic DNA level by using screening techniques limited to exons and canonical splice sites at the exon boundaries [19]. We observed 21 patients who had apparently no *OA1 *gene mutations or deletions despite presenting an apparently X-linked form of ocular albinism. These patients may have this type of intron mutation or may have mutations that alter the regulatory regions of the gene, such as the promoter region, silencers or enhancers.

Normally, with the techniques currently used for the molecular genetics diagnosis of X-linked ocular albinism, one third of affected individuals is not found to carry such mutations [10]. Other patients have a completely or partially deleted gene.

Apparently sporadic cases may be due to several reasons. In some instances, we could simply not be able to get all the DNA samples of the families. Sometimes, *de novo *germinal alterations could have arisen, the mechanisms of which are diverse, including DNA lesions and repair or recombinations during meiosis [21]. The latter mechanism looks more plausible, as there may be many repeated sequences within introns or around the gene, leading to misalignments of X chromosomes during meiosis and unequal crossing-over(s). This may explain the high deletion rate in *OA1 *gene. Germinal mosaicism may also be a cause of sporadic deletion in *OA1*.

Another possibility is that some of these patients may have been considered erroneously as affected by X-linked ocular albinism while they are in fact affected by very mild forms of oculocutaneous albinism. The limited size of the pedigrees analyzed in this study prevents us from stating with certainty the mode of inheritance of all the clinically diagnosed ocular albinisms included in our cohort. We cannot rule out that ocular albinism may constitute a heterogenous genetic ensemble despite being apparently clinically homogenous. However, in light of all the studies published so far, this hypothesis is very unlikely and all clinical forms of ocular albinism appear with a very high degree of probability linked to genetic alterations of the OA1 gene, despite the variability of symptoms observed in different patients [22].

Studying the size and the sequences of the mRNA from genes encoding proteins involved in melanogenesis is hindered by the extreme difficulty in their amplification by RT-PCR of total RNA extracted from lymphoblastoid or fibroblast cell lines from affected patients. The illegitimate transcription of such cell lines to obtain cDNA corresponding to the transcripts encoding melanogenic proteins has never been shown to be particularly efficient. Indeed, these genes either are not transcribed or are only weakly transcribed in these cell lines. Therefore, we would need to obtain skin biopsies from affected patients, purify melanocytes from these samples, culture them and then extract total RNA from these cultures before carrying out RT-PCR.

Our mutational screening would certainly benefit from quantitative genomic PCR [23] in order to increase the probability of detecting other *OA1 *mutations that may have gone unnoticed by the sequencing procedure used for small, amplified exon fragments studied in this report.

## Conclusion

Our results highlight the role of key amino-acids in both OA1 structure and function, as well as the need for detailed study of the molecular mechanisms of the splice site mutations and their consequences both at the mRNA and protein levels. The search for additional intronic or extragenic *OA1*genetic alterations, including deletions, remains an important goal for future molecular investigations of X-linked recessive ocular albinism. However, all forms of clinically diagnosed cases of apparently ocular albinism are not necessarily X-linked recessive forms of ocular albinism. This may explain, at least partially, the apparently low level of molecular genetic alterations detected by screening the *OA1 *gene in patients affected by ocular albinism.

## Competing interests

The author(s) declare that they have no competing interests.

## Authors' contributions

HM sequenced the patients' DNA and drafted the manuscript, CV and DM helped for the experiments, CJ helped drafting the manuscript, HD, DB, FM, DS, AL, JS, EH, DL, ES, EM, JK, OR, JLD and MA provided ophthalmic diagnosis, supplied patient DNA and participated in the editing of the manuscript. CM, MM and MA designed and supervised the study. MA also helped drafting the manuscript. All authors read and approved the final manuscript.

## Pre-publication history

The pre-publication history for this paper can be accessed here:


